# FeniVerse: A parallel corpus of Feni dialect, standard Bengali, and English

**DOI:** 10.1016/j.dib.2025.112250

**Published:** 2025-11-07

**Authors:** Mehraj Hossain Mahi, Anzir Rahman Khan, Zesanul Hoque, Mayen Uddin Mojumdar

**Affiliations:** Multidisciplinary Action Research Lab, Department of Computer Science and Engineering, Daffodil International University, Birulia, Dhaka 1216, Bangladesh

**Keywords:** Feni Dialect, Bangla language, Natural language processing, Bangla language identification, Dialects identification, Bangla regional dialects

## Abstract

FeniVerse is a trilingual parallel corpus comprising 4094 entries in English, Standard Bangla, and the Feni Dialect, totaling 12,282 sentence-aligned translations. It addresses the scarcity of datasets and tools for Bangla dialects, particularly the underrepresented Feni Dialect, spoken by approximately 1.6 million people in southeastern Bangladesh. As the first dataset for this dialect, FeniVerse is a valuable resource for computational linguistics. The corpus is manually curated, with each entry cross-checked by native Feni speakers and regional volunteers to ensure accuracy while preserving phonological, lexical, and syntactic variations. FeniVerse is openly accessible and designed for seamless integration into NLP (Natural Language Processing) pipelines. Existing AI and NLP models struggle to process the Feni Dialect due to limited training data, making this dataset critical for developing effective language technologies. The proposed dataset supports diverse applications, including dialect identification, machine translation, and cross-linguistic analysis, while promoting equitable AI development for low-resource languages. By introducing the first trilingual dataset for the Feni Dialect, FeniVerse contributes to computational linguistics and the digital preservation of regional Bangla dialects, supporting both academic research and practical AI applications.

Specifications Table

**Table:** 

Subject	Computer Sciences
Specific subject area	Feni Dialect, Natural Language Processing, Bangla text classification, Low-Resource Languages
Type of data	Table (text/string)
Data collection?	FeniVerse was created through interviews with native Feni speakers, who provided sentences in their dialect aligned with Standard Bangla and English. All participants gave informed consent, and the data were transcribed and then verified
Data source location	The list of the primary data sources used to create the FeniVerse dataset is as follows. Primary data sources: Feni Sadar, Feni (Latitude: 23.0159° N, Longitude: 91.3976° E) Daganbhuiyan, Feni (Latitude: 22.9353° N, Longitude: 91.3032° E) Chhagalnaiya, Feni (Latitude: 23.0248° N, Longitude: 91.5146° E) Sonagazi, Feni (Latitude: 22.8576° N, Longitude: 91.3956° E) Parshuram, Feni (Latitude: 23.2151° N, Longitude: 91.4437° E) Fulgazi, Feni (Latitude: 23.1520° N, Longitude: 91.4213° E)
Data accessibility	Repository name: Mendeley Data Data identification number: 10.17632/25r923frfj.1 Direct URL to data: FeniVerse: A Parallel Corpus of Feni Dialect, Standard Bengali, and English

## 1 Value of the Data

• FeniVerse is the first dataset ever created for the Feni Dialect, providing a pioneering resource for Bangla dialectal research.
• It is the first trilingual parallel corpus including English, Standard Bangla, and Feni Dialect, filling a critical gap in resources for Bangla dialects.
• The dataset enables comparative linguistic and sociolinguistic studies, allowing analysis of grammar and vocabulary differences between Standard Bangla and Feni.
• For NLP and AI applications, FeniVerse supports dialect classification, machine translation, and cross-linguistic modelling. This dataset enables the training of a classifier to distinguish Feni Dialect from Standard Bangla, a key step for building dialect-sensitive NLP tools.
• Its trilingual alignment makes it particularly valuable for building and evaluating dialect-to-standard Bangla and dialect-to-English translation systems, with practical applications in education, communication, and cultural preservation.
• Incorporating an underrepresented dialect improves fairness and inclusivity in NLP, reducing bias toward Standard Bangla and supporting more balanced language technologies.
• Despite advances in AI and NLP, existing models still struggle to understand the Feni Dialect, highlighting the importance of FeniVerse in enabling AI to process and learn this underrepresented language variety.
• The dataset is open, reusable, and extendable, making it a versatile resource for researchers in linguistics, and artificial intelligence worldwide.

## 2 Background

Bangla (Bengali) is the fifth most spoken language in the world [1]. While Standard Bangla dominates in education, administration, and media, the country’s 64 districts are home to >55 regional languages and dialects [2] that reflects a high degree of linguistic diversity. Among these, the Feni Dialect which is spoken by around 1.6 million people is one of the most distinctive dialect in south-eastern Bangladesh [3]. It differs significantly from Standard Bangla in vocabulary, pronunciation, and syntax, making it challenging for many native Bangla speakers to understand [4]. Despite its cultural and linguistic importance, computational resources for Feni dialects remain scarce. To address this gap, we present FeniVerse, a trilingual parallel corpus containing aligned text in English, Standard Bangla, and Feni Dialect.

## 3 Data Description

**Fig. 1:**
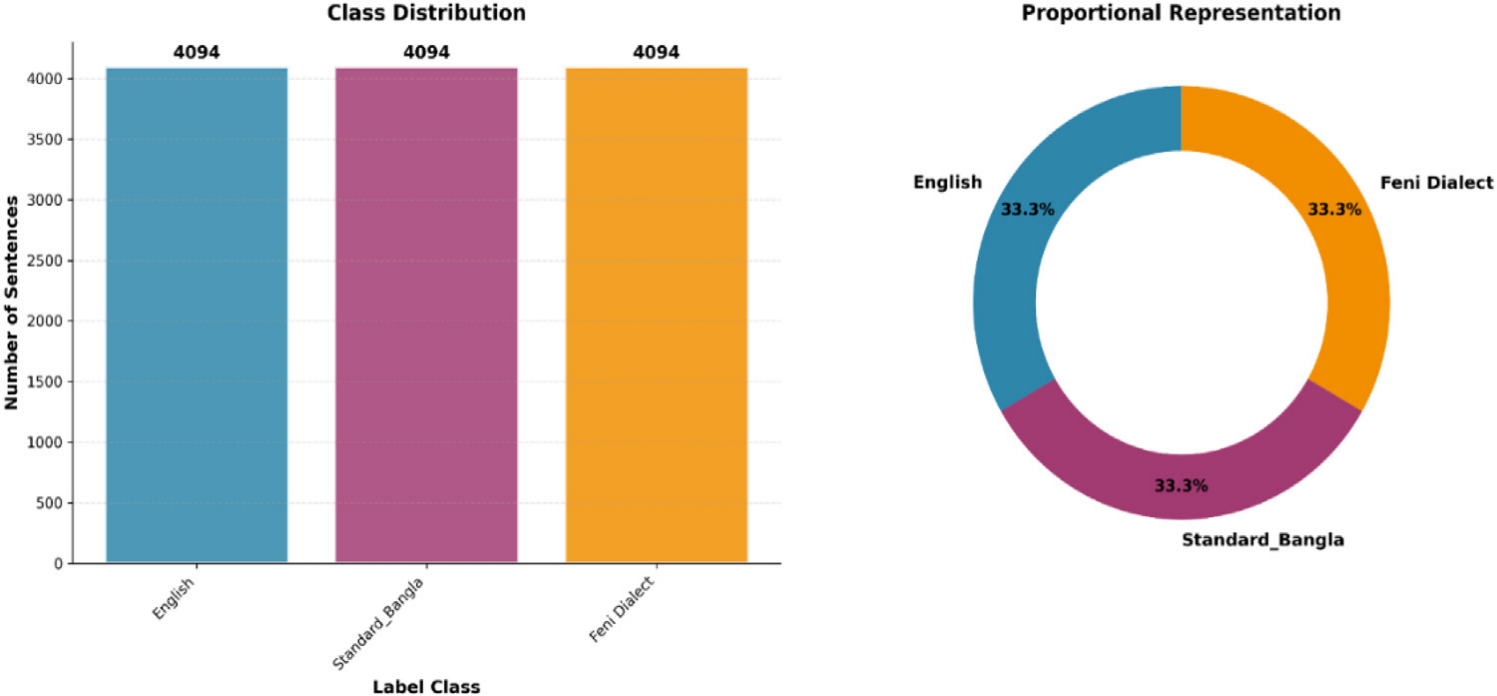
Dataset class wise description.

We have made our datasets openly accessible on Mendeley data repository [5]. The dataset is a trilingual parallel corpus containing aligned text in English, Standard Bangla, and Feni Dialect. The dataset is organized into three columns - one for each language and contains 4094 sentences per language, resulting in a total of 12,282 sentences across all three languages. Each row in the dataset represents a sentence-level alignment, where the English sentence corresponds to its translations in Standard Bangla and the Feni Dialect. The dataset is stored in CSV format. Fig. 1 shows the dataset class-wise description and the percentage of data in each class.

The Feni Dialect text preserves regional vocabulary, syntactic patterns, and phonological variations that distinguish it from Standard Bangla and an authentic representation of local language use. The dataset covers a diverse range of topics, ensuring broad applicability for both linguistic research and computational experiments.

**Fig. 2:**
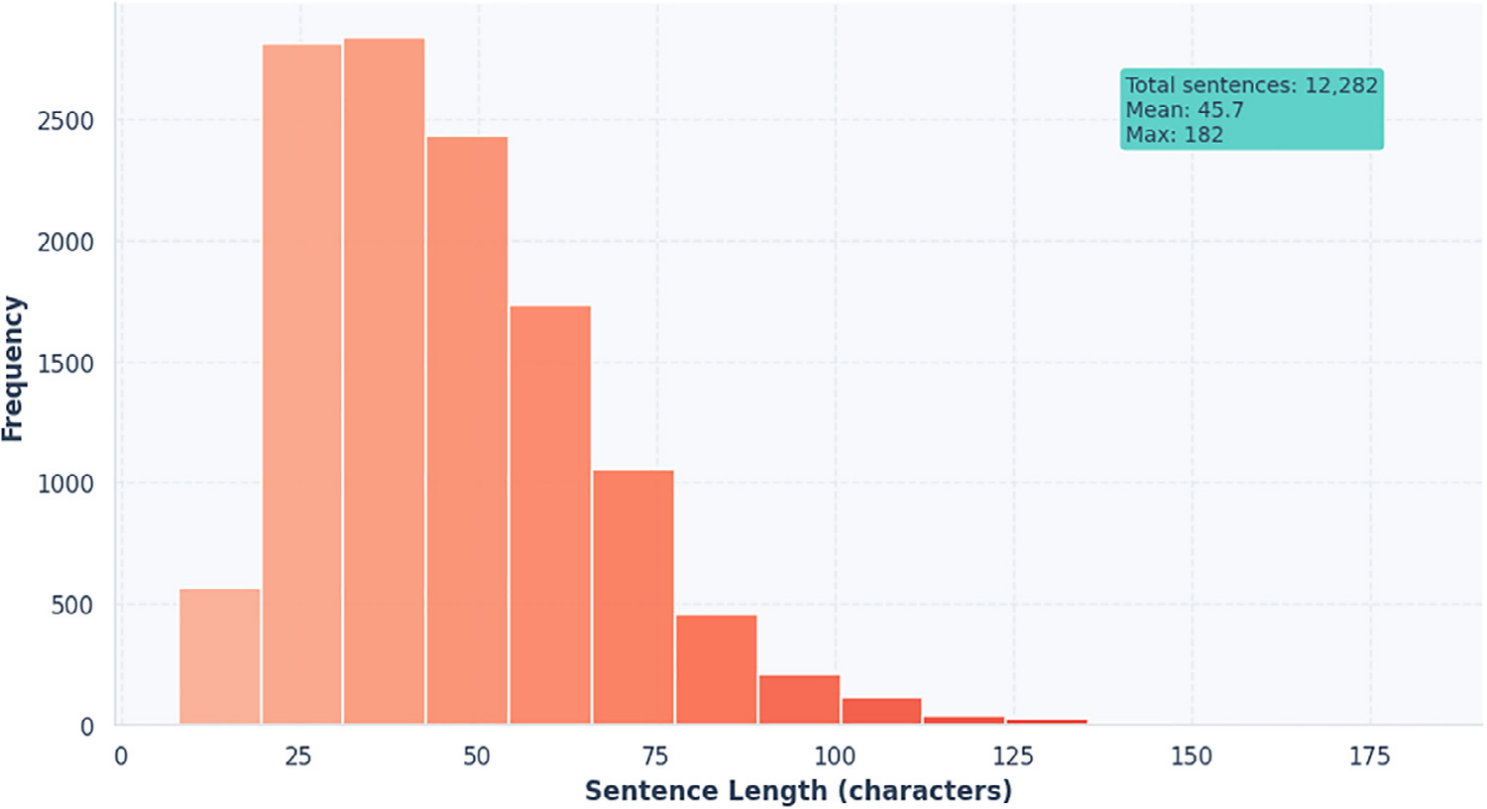
Histogram of sentence lengths (in characters).

Table 1 presents sample entries from the dataset, showing the trilingual representation of sentences across English, Standard Bangla, and Feni Dialect, providing an overall view of the dataset’s content. Fig. 2 shows the distribution of sentence lengths (in characters) across the dataset. Table 2 Summary of word and character counts per sentence for each language/dialect in the FeniVerse dataset, showing maximum, minimum, and average values.

**Table 1:** Example of the dataset. The data are stored in a CSV file with the following columns:.

**Table 2:** Summary statistics of sentence lengths in words and characters across languages.

Class	Word Count	Max_words	Min_words	Avg_words	Max_chars	Min_chars	Avg_chars
**English**	36,663	34.0	2.0	8.955300	182.0	8.0	47.826087
**Standard_Bangla**	30,790	24.0	2.0	7.520762	135.0	10.0	44.260381
**Feni Dialect**	31,003	25.0	2.0	7.572789	134.0	9.0	45.048608

**Fig. 3:**
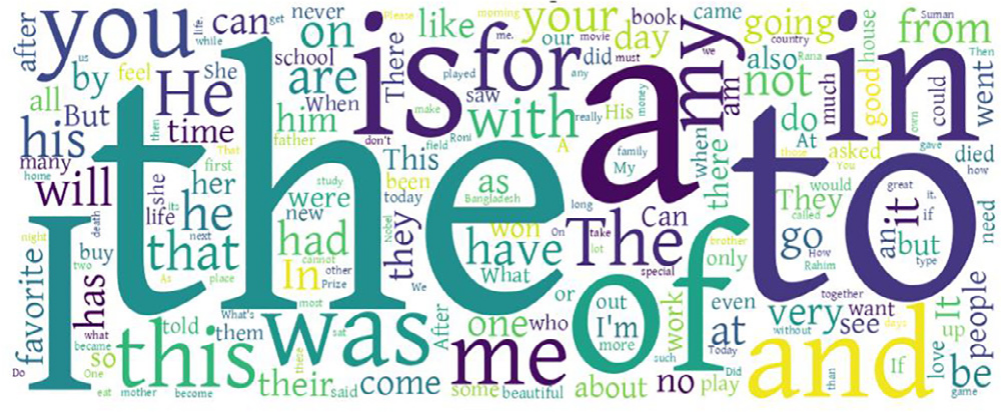
Word cloud for English.

**Fig. 4:**
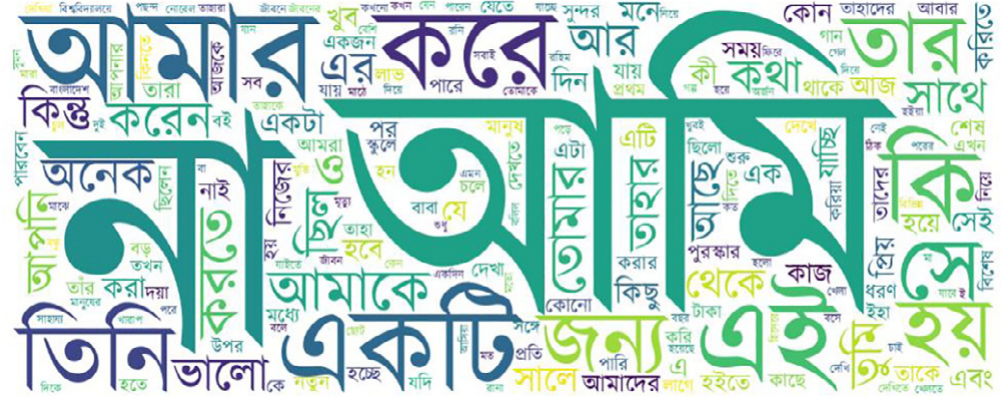
Word cloud for Standard Bangla.

**Fig. 5:**
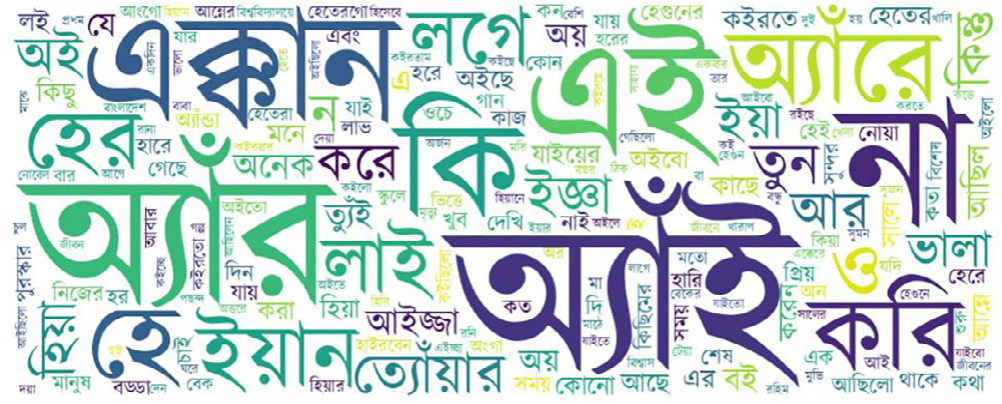
Word cloud for Feni Dialect.

To visually depict the most frequently used words across each language and dialect, Fig. 3, Fig. 4, Fig. 5 present a series of word clouds. These graphics offer a comparative view of the distinctive lexical patterns and linguistic tendencies present in each variety. Prior to creating the word clouds, multiple pre-processing steps were applied using the Python Pandas library: normalization of letters, removal of numbers, and punctuation. Sentences were then grouped according to their label and merged into unified text blocks using the str.cat() function in the pandas library. Common stop words were also removed to enhance clarity. The word clouds were generated using Python’s Word Cloud library without applying any predefined frequency limits. As a result, these visualizations highlight the most salient and recurrent words within each language or dialect category. Importantly, the most frequent words shown are consistent with the expected vocabulary of a daily-use corpus, matching the vocabulary of each language and dialect, which helps validate the data's content and its representation of everyday communication captured in the FeniVerse dataset.

Table 3 provides a comparative overview of the FeniVerse dataset alongside other Bangla language dialect datasets. It highlights that no previous dataset includes the Feni Dialect which makes FeniVerse the first trilingual, open-access, manually curated, and sentence-aligned resource. The table also lists key features of other Bengali regional datasets to emphasize FeniVerse’s unique contribution in terms of dialectal coverage and dataset size.

**Table 3:** Comparison with existing bangla language datasets.

Dataset	Classes	Dataset type	Dataset Size	English Includes	Feni Dialect
ONUBAD [6]	5	Text	3920		
MELD [7]	4	Text	3046		
Our Dataset FeniVerse	3	Text	12,282		

## 4 Experimental Design, Materials and Methods

### 4.1 Methodology

**Fig. 6:**
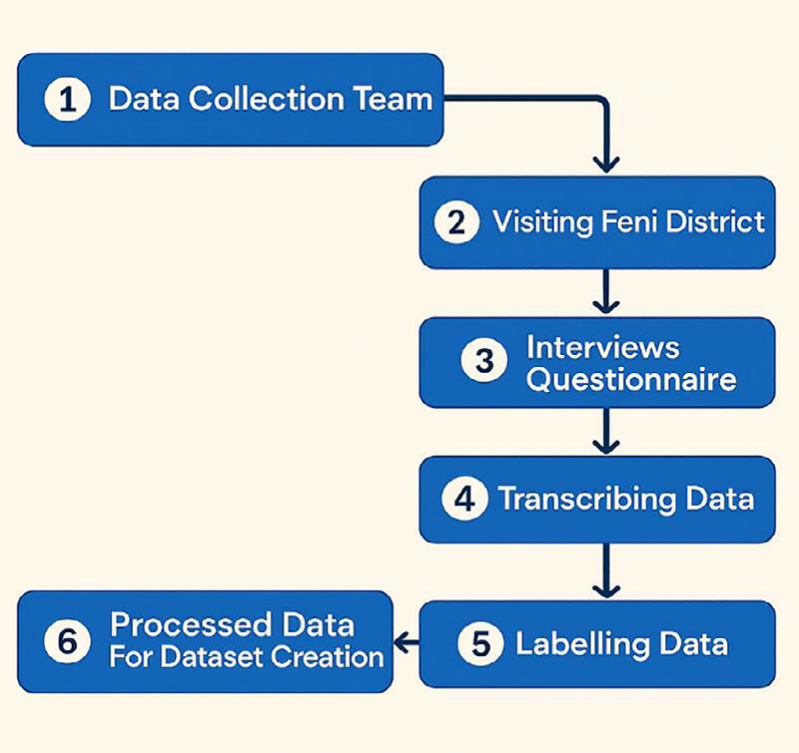
Data collection process.

Fig 6. depicts the workflow for FeniVerse dataset creation. The process begins with announcing for recruiting a volunteering team that consists of Feni native students from MAARS(Multidisciplinary Action Research Lab) . Feni native students were selected for the data collection team. Then the data collection team visited the Feni district to conduct interviews and distribute questionnaires. The process went smoothly as the team consisted of Feni native resident. Collected responses were transcribed, labeled, and processed to generate the final dataset, ensuring structured and accurate representation of the Feni Dialect. All the sentences are from simple, everyday conversations and were manually aligned using Microsoft Excel.

1. **Data Collection Team:** A dedicated data collection team, including the 4 native Feni students was formed to initiate the process. This team was responsible for planning, managing, and executing the fieldwork activities. Their role was to ensure that the research objectives were met by following a systematic and organized approach**.**
2. **Visiting Feni District:** Once the team was ready, they visited the Feni district with specific towns. This visit allowed the researchers to directly interact with the local population, observe the environment, and create a real connection with the participants.
3. **Interviews and Questionnaires:** Data samples were collected through structured interviews and written contributions from individuals aged 20 to 78. The dataset encompasses everyday sentences covering a wide range of topics, reflecting conversations and experiences across various aspects of daily life, including work, family, social interactions, culture, and personal interests. Volunteers acted as translators during the interviews, facilitating communication and transcribing responses from native speakers into written form. Standard Bengali texts were also included to enable comparison between regional dialects and the standard language. Additionally, English translations were provided to expand the dataset’s accessibility for international researchers, educators, and speakers who do not understand Bengali.
4. **Transcribing Data:** All spoken responses were carefully transcribed into written form with the help of the native volunteers. This process ensured the preservation of the raw information in a structured manner while reducing the chances of misinterpretation or data loss.
5. **Labelling Data:** After transcription, the dataset was systematically labelled and categorized according to linguistic. This step ensured organization and made the dataset more accessible for further analysis.
6. **Processed Data for Dataset Creation:** The final stage involved processing the labelled data into a structured dataset. To ensure accuracy and reliability, a subset of the collected samples (1365 * 3) underwent verification by additional native speakers from the communities we visited. Any discrepancies identified during this process were addressed through peer review and consensus-building sessions with native-speaking university volunteers. In cases of disagreement, the reviewers discussed the discrepancies and reached a consensus, often consulting with a third reviewer. Furthermore, each data sample was cross-verified by at least two speakers of the respective language, which helped maintain consistency and minimize potential transliteration errors. This rigorous verification step strengthened the overall quality and trustworthiness of the dataset.

In summary, the FeniVerse dataset was created through a carefully designed and systematic process, from field data collection to transcription, labelling, and verification. The methodology ensured high-quality, accurate, and representative data of the Feni Dialect, while maintaining ethical standards and participant confidentiality.

**Fig. 7:**
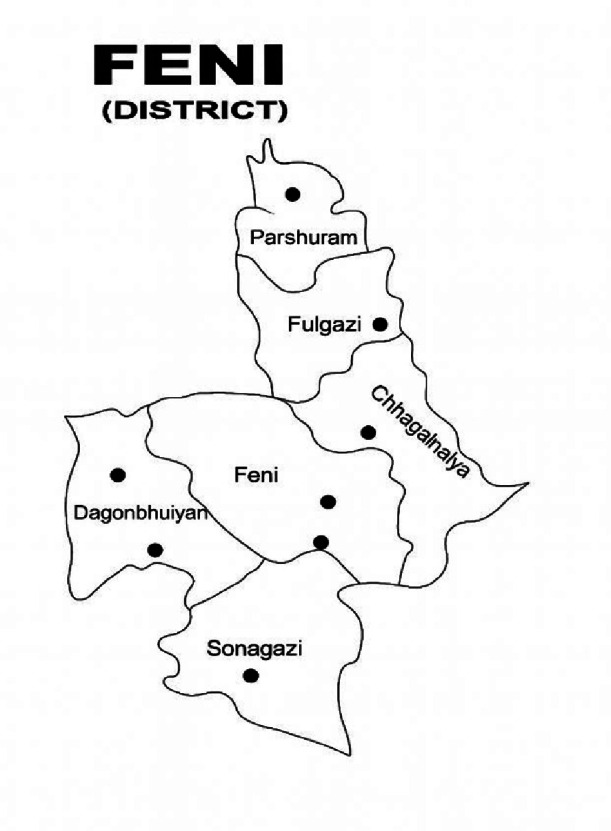
Geographical location of Feni District with Upazila boundaries.

Fig. 7 shows the geographic location of the Feni community in southeastern Bangladesh, indicating the area where the Feni Dialect was collected. The map illustrates the localized distribution of Feni speakers, forming a key part of the trilingual dataset.

**Fig. 8:**
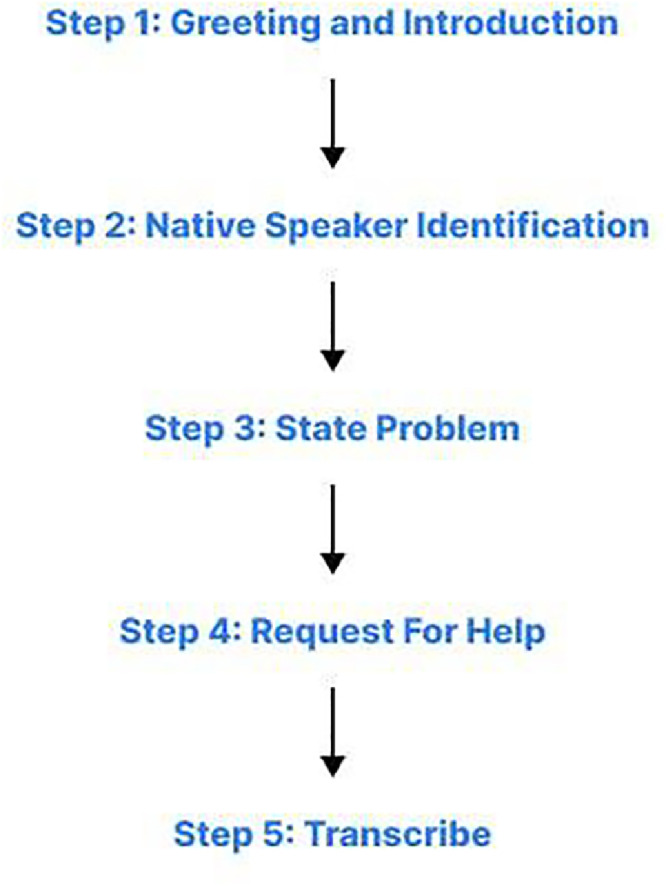
Process of interview.

As described in Fig. 8, we conducted interviews using themes such as daily conversations, cultural expressions, greetings, and everyday interactions. We asked participants open-ended questions like:

• “How do you say ‘What are you doing?’ in your language?”
• “What do you usually say when meeting a friend?”
• “Can you share a common household phrase or greeting?”

The participants provided responses in their native Feni language, and we meticulously recorded their exact sentences to include in our dataset. This method guaranteed the inclusion of genuine, naturally occurring language samples.]

## Limitations

While FeniVerse is a valuable resource for Bengali dialectal analysis, it has certain limitations:

1. The dataset contains 4094 sentences per language (12,282 total), which may be insufficient for training very large-scale NLP models, though it is adequate for many linguistic and computational tasks.
2. All sentences are manually curated and aligned, ensuring quality, but this limits the overall dataset size; future expansions could enhance coverage.
3. The dataset focuses exclusively on the Feni Dialect and does not capture the full diversity of the 55+ regional dialects of Bangladesh.
4. Being text-only, it lacks speech, audio, or phonetic annotations, which are important for speech recognition and phonological research. This presents an opportunity for future work to create a complementary spoken corpus of the Feni Dialect.
5. While translations are aligned across English, Standard Bangla, and Feni, idiomatic or culturally specific expressions may not always have direct equivalents. Notably, some two-word phrases in Standard Bangla may appear as a single word in Feni, which can introduce minor semantic differences.

These limitations highlight several opportunities for future work, such as expanding the dataset to accommodate larger-scale NLP models, incorporating additional regional dialects, creating a complementary spoken corpus of the Feni Dialect, and refining idiomatic translation alignments.

## Ethics Statement

The authors confirm that the creation and release of the FeniVerse dataset adhere to strict ethical standards. All data have been fully anonymized, ensuring that no personally identifiable information is included. Data collection and redistribution comply with all relevant legal and institutional policies. All contributors were informed that participation in the dataset creation was entirely voluntary and that no academic credit or incentives would be provided. Informed consent was obtained from all participants prior to data collection. The dataset is intended solely for research purposes, and care has been taken to minimize any potential risks to contributors. This study was conducted following the ethical guidelines of Daffodil International University Research Ethics Community(REC), and the research protocol was approved under Ethics Protocol Number Ref:DIU/Dean/FSIT/ 2025–4155.

## Credit Author Statement

**Mehraj Hossain Mahi:** Data curation, Validation, Methodology, Visualization, Conceptualization, Writing–original draft; **Anzir Rahman Khan:** Data curation, Validation, Visualization, Writing–review & editing; **Zesanul Hoque:** Data curation, Validation; **Mayen Uddin Mojumdar:** Validation, Supervision.

## Declaration of generative AI and AI-Assisted technologies in the writing process

During the preparation of this manuscript, the authors utilized AI to assist in improving the language. The content was subsequently reviewed and revised by the authors, who take full responsibility for the final manuscript.

## Data Availability

Mendeley DataFeniVerse: A Parallel Corpus of Feni Dialect, Standard Bengali, and English (Original data).

## Appendix Supplementary materials

Image, application 1

Image, application 2
