# Supplementary material for: FeniVerse: A parallel corpus of Feni dialect, standard Bengali, and English
Source: Data Brief. 2025 Nov 7;63:112250. doi: 10.1016/j.dib.2025.112250 (PMC12666053; doi:10.1016/j.dib.2025.112250)
Supplement: Supplementary file 1 [file mmc1.pdf]

Supplementary File: Data Collection Procedure for Feni Dialect Interviews

Overview

This supplementary document explains the detailed process used to collect spoken data in the Feni dialect through interviews. The goal was to record authentic local speech, translate it into Standard Bangla, and prepare it for linguistic and computational analysis. All interviews were conducted naturally in Bangla and Feni dialect. Participants were chosen based on their residence and long-term exposure to the Feni region, ensuring genuine dialect use. The spoken data in the Feni dialect were collected through interviews conducted in a manner that ensured the anonymity of the participants. To protect their privacy, all participants were not identified by name, and personal information was excluded from the dataset

Step 1: Greeting and Introduction

Each interview began with a friendly and polite conversation to make the participant feel comfortable. The interviewer explained the research purpose and requested permission to record their responses.

Speaker	Bangla (Standard)	English Translation
Interviewer	ভাই/আপা, একটু কথা বলার সময় হবে?	Excuse me, do you have a moment to talk?
Participant	হ্যাঁ, বলেন।	Yes, please go ahead.
Interviewer	আমরা ফেনী এলাকার ভাষা নিয়ে গবেষণা করছি। আপনি কি ফেনীতেই জন্মেছেন?	We are conducting research on the Feni dialect. Were you born in Feni?
Participant	হ্যাঁ, আমি ফেনীর ছাগলনাইয়া থানা থেকে।	Yes, I'm from Chhagalnaiya, Feni.

Step 2: Feni Dialect Identification

The interviewer confirmed regional background and language familiarity.

Demographic Information (জনসংখ্যাগত তথ্য):

1. Age (বয়স): ______
2. Gender (লিঙ্গ):
   - Male (পুরুষ)
   - Female (মহিলা)

- Other (অন্যান্য)

3. Place of Residence (বাসস্থান - গ্রাম/শহরের নাম উল্লেখ করুন): ____________________
4. How long have you lived in the Feni region? (আপনি কতদিন ধরে ফেনী অঞ্চলে বসবাস করছেন?)
    - Less than 5 years (৫ বছরের কম)
    - 5–10 years (৫–১০ বছর)
    - More than 10 years (১০ বছরের বেশি)

- 5. Does everyone in your family speak the Feni dialect? (আপনার বাড়িতে সবাই কি ফেনীর ভাষায় কথা বলে?)

Step 3: Explain the Research Purpose

The interviewer clearly explained the objective of the research — to understand and document the local speech style and its translation into Standard Bangla.

Speaker	Bangla (Standard)	English Translation
Interviewer	আমাদের লক্ষ্য হলো ফেনী এলাকার ভাষা কেমন, সেটা বিশ্লেষণ করা।	Our goal is to analyze how people speak in the Feni region.
Interviewer	এজন্য আমরা কিছু সাধারণ বাক্য আপনার ভাষায় জানতে চাই।	So, we'd like you to say a few sentences in your dialect.

Step 4: Request for Help

Once the participant agreed, the interviewer asked them to help by saying or translating simple Bangla sentences into the Feni dialect.

Speaker	Bangla (Standard)	English Translation
Interviewer	আপনি কি আমাদের সাহায্য করতে পারবেন, কিছু বাক্য ফেনীর ভাষায় বলে?	Can you help us by saying some sentences in Feni dialect regarding this topic?

Step 5: Translation and Transcription

Participants were given common Bangla sentences related to daily life. They were asked to say the same sentences naturally in their Feni dialect. The responses were recorded and later transcribed manually, followed by Standard Bangla translation and English gloss.

No.	Standard Bangla Sentence	Feni Dialect Version	English Gloss

1	আমি স্কুলে যাচ্ছি।	অ্যাঁই স্কুলে যাইয়ের।	I am going to school.
2	ওখানে বৃষ্টি হয়েছে নাকি?	হিয়েনে বৃষ্টি অইছে নিকি??	Has it rained there?
3	কুকুরটি মারা গেছে	কুত্তা ইয়া মরি গেছে	The dog is dead
4	তারা বিয়ে করেছে	হেগুনে বিয়া কইচ্ছে	They got married

Data Organization and Verification

It contains all verified transcriptions, translations, and alignment files in two main formats — **CSV** and **XLSX** — to ensure compatibility with both linguistic analysis tools and spreadsheet software.

All data were cross-checked by multiple reviewers for linguistic consistency.

Ethical Considerations

Before interviews, each participant received a verbal explanation about the research and data use. Consent for recording was taken verbally and respected at all times. No personal names or identifiable information were published.

Conclusion

This structured Feni dialect-based interview method ensured natural conversation, accurate regional representation, and reliable translation for linguistic research. The five-step process — Greeting, Dialect Identification, Explain, Request, and Transcribe — provided a consistent and ethical way to collect authentic Feni speech data.
