# Supplementary material for: FeniVerse: A parallel corpus of Feni dialect, standard Bengali, and English
Source: Data Brief. 2025 Nov 7;63:112250. doi: 10.1016/j.dib.2025.112250 (PMC12666053; doi:10.1016/j.dib.2025.112250)
Supplement: Supplementary file 2 [file mmc2.pdf]

// Acknowledgements

We would like to extend our heartfelt gratitude to the following volunteers for their invaluable support and dedication throughout this project

Information of Volunteers

Name	Language	Regional Dialects	Age	Gender	Education Level	Locations
Md. Khaledujjaman	Bangla	Feni Dialect	25	Male	B.S.c in MCT	Feni
Muhtasimul Hoque Arnab	Bangla	Feni Dialect	25	Male	B.B.A in THM	Feni
Abdur Rahim	Bangla	Feni Dialect	23	Male	Diploma in CSE	Feni
Sunjimul Hoque Ayon	Bangla	Feni Dialect	25	Male	B.B.A in THM	Feni

Information of Additional Native speakers that helped in further verification:

Name	Language	Regional Dialects	Age	Gender	Education Level	Locations
Md Abu Tayub	Bangla	Feni Dialect	24	Male	Undergraduate	Feni
Imrul Kayes	Bangla	Feni Dialect	25	Male	Undergraduate	Feni
Abdullah Al Azad	Bangla	Feni Dialect	24	Male	Undergraduate	Feni

Their diverse perspectives, sincere efforts, and unwavering enthusiasm significantly contributed to the success of this work. We are truly grateful for their commitment and valuable contributions. All individuals listed above served purely in a voluntary capacity to assist with data collection.
